# H″IT″ting the Barriers for Exercising during Social Isolation

**DOI:** 10.3390/biology9090245

**Published:** 2020-08-24

**Authors:** Daniel Souza, Victor Coswig, Claudio Andre Barbosa de Lira, Paulo Gentil

**Affiliations:** 1 College of Physical Education and Dance, Federal University of Goiás, Goiânia 74690-900, Brazil; daniel_souza86@hotmail.com (D.S.); andre.claudio@gmail.com (C.A.B.d.L.); 2 College of Physical Education, Federal University of Pará, Castanhal 68746-360, Brazil; vcoswig@gmail.com; 3 Hypertension League, Federal University of Goiás, Goiania 74605-020, Brazil

**Keywords:** COVID-19, aerobic exercise, immunity, high-intensity interval training, calisthenics, intermittent exercise

## Abstract

Aerobic exercise is traditionally recommended to improve general health and prevent many non-communicable diseases. However, the measures adopted to control the novel Coronavirus (COVID-19) outbreak culminated with closing of exercise facilities and fitness centers and, as a primary consequence, impaired aerobic exercise practice. This contributed to an increase in risk factors associated with physical inactivity such as insulin resistance, high blood pressure, low-grade inflammation, weight gain, and mental health problems. The scenario is worrisome, and it is important to propose alternatives for exercise practice during the COVID-19 pandemic. Interval training (IT) emerges as an exercise mode that might be feasible, low-cost, and potentially safe to be performed in many different places. IT consists of interspersing relative brief bouts of high-intensity exercise with recovery periods and promotes similar or greater health benefits when compared to moderate-intensity continuous exercise. Among the different types of IT, sprint interval training and “Tabata protocols” might be particularly useful during social isolation. These protocols can be controlled and performed without the need of complex equipment and can be adapted to different places, including domestic environments. In this article, we present variations of IT as possible alternatives to cope physical inactivity during COVID-19 pandemics with a focus on its practical applications. The protocols suggested can be performed without the need of specialized equipment or facilities, in a time-efficient manner, and aiming to prevent detraining or even improve physical fitness and general health.

## 1. The Problem

Regular aerobic exercise is recommended by health organizations and exercise specialists for disease prevention, healthy aging and reducing mortality rates [1,2,3]. These benefits have been associated with increases in cardiorespiratory fitness (VO_2_max), weight loss and improvements to the immune system [4,5,6,7]. Aerobic training may be performed using different types of exercises (e.g., jogging, bicycle, running, rowing, swimming), with or without specialized equipment and in many different places, such as gyms, health clubs, outdoors or public spaces. The outcomes derived from an aerobic training program seem be dependent more on adequate exercise prescription than on the equipment or environment [8], with a special attention to adequate intensity control [9]. The importance of intensity in achieving functional and health related outcomes has been evidenced in different groups, where high-intensity training provided similar or greater benefits than moderate-intensity exercise [10,11,12,13,14].

The practice of aerobic training might be compromised during the novel Coronavirus (COVID-19) outbreak due to the preventive measures adopted by governments, such as social isolation and avoidance of closed spaces and agglomeration. This might lead to a reduction in physical activity [15] and increase in sedentary behavior, which might contribute to increase the risk for cardiometabolic and infectious diseases, resulting in increased risk for morbi-mortality [16,17,18,19]. The cessation of aerobic training might lead to adverse health effects such as decreases in cardiorespiratory fitness [20], impaired insulin function [21], weight gain [22,23], autonomic imbalance [24] and functional decline [25]. The current pandemic scenario also potentially contributes to exacerbate mental health problems, such as mood disorders, panic, perceived stress, anxiety and depressive symptoms [26,27,28,29]. Moreover, it has been shown that public health measures adopted to control coronavirus dissemination may negatively impact well-being of inactive individuals [30].

In most countries adopting social distancing, exercise facilities, such as gyms and fitness centers, are closed or under heavy restrictions, which naturally limit exercise practice. Although outdoor exercise was allowed, or even recommended, the heavy breathing during cycling, jogging or running might contribute to increase droplet trail [31]. These findings suggest that the performance of conventional aerobic training in public spaces or running tracks should observe adequate social distancing. Therefore, it is important to understand how to appropriately exercise in both outdoor and indoor environments without the need of specialized equipment to provide safe and viable approaches to exercise during social isolation [16,18,32].

Considering the health benefits associated to aerobic activity, it is important to propose training strategies that can provide significant improvements in physical fitness and mental health while adapting to the social restrictions measures due to the coronavirus outbreak. Independent of the restrictive measures, many people might be afraid of exercising in public spaces, gyms, fitness center or even running tracks due the risk of contamination and there is also a real possibility of a second wave of COVID-19 [33]. Accessibility and cost may be considered as additional barriers to the regular practice of physical exercise [34,35]. Therefore, it is important to provide options for exercise practice that are adaptable to different situations and can overcome perceived barriers.

When analyzing the current situation, interval training (IT) emerges as an interesting option. IT consists of interspacing periods of intense exercise with periods of low-intensity exercise or passive recovery. This type of exercise has been increasingly recognized as being efficient to promote metabolic and cardiorespiratory adaptations [36,37,38,39]. IT can be performed in different forms, with different modes of intensity control such as perceived effort, heart rate, mechanical loading and intensity associated to VO_2_max [40]. In addition, IT might involve exercise modalities that require minimal or affordable equipment like cycling, running, calisthenics, battling hope and stair climbing [41,42]. This wide range of possibilities makes IT highly adaptable, which might be particularly interesting at the current time. However, some questions have to be raised about its feasibility and safety, especially considering the impact of high-intensity efforts on the immune system and infection risk [43,44], in particular upper respiratory tract infection (URTI) [26].

The present article discusses IT variations that could be feasible, suitable, low cost, and safe for maintaining/improving physical fitness and general health during COVID-19 outbreak.

## 2. Interval Training Characteristics and Health Benefits

Due to the intermittent characteristic, IT allows to accumulate a higher volume of vigorous exercise [45] and higher time close to or at maximal oxygen consumption levels when compared to continuous exercise [40]. Evidence from healthy and clinical populations have consistently shown that IT promotes metabolic and cardiorespiratory adaptations of similar or even greater magnitude than higher volumes of moderate-intensity continuous exercise [36,37,38,39]. Furthermore, IT is considered effective to reduce cardiometabolic risk factors associated with increased mortality and morbidity, such as high blood pressure [46], excessive body fat [47], impaired glucose metabolism [48], chronic low-grade inflammation [49] among others. Therefore, considering that people with arterial hypertension, overweight/obesity, and diabetes mellitus might be at higher risk for severe illness from COVID-19 [50], it is reasonable to assume that IT might be an interesting strategy to mitigate this risk.

In addition to its physiological benefits, IT emerges as a promising non-pharmacological strategy to manage mental health problems, such as depression and anxiety [51,52,53]. The psychological benefits provided by IT might be equivalent to those achieved with traditional aerobic training during rehabilitation [51] and in the management of major depressive disorders [52]. It is important to point out that the impact of physical exercise on mental health seems be dependent on the level of symptoms, suggesting that individuals with more severe symptoms might benefit more from exercise performance [54].

IT can be performed in different ways and with different methods for controlling intensity [40,42]. Among them, sprint interval training (SIT) and “Tabata” protocols might be particularly interesting during the COVID-19 pandemic. These methods do not require complex testing, can be performed in a wide range of situations (equipment and spaces) and their intensity can be easily controlled.

SIT is a specific type of IT that involves relative short bouts (≤30 s) of maximal or “all-out” efforts [40]. SIT became popular in recent decades with the Wingate-based protocol (4–6 × 30 s “all-out” sprints interspersed with 4-min recovery) [36]. Whilst this variation requires specialized equipment and high motivation, SIT can be adapted in a diversity of ways and modalities to be performed in different places and with less discomfort [55,56].

There is evidence that SIT protocols might even more time efficient. Previous studies showed that 3 × 20 s cycling sprints improved cardiorespiratory fitness and glucose metabolism in inactive and obese adults [55,57]. A previous meta-analysis concluded that as little as two sprints might be recommended to increase VO_2_max [58]. This very low volume SIT may be spread throughout the day, what has been called “sprint snacks” [59], and performed in a practical manner, such as using stair climbing [60].

The performance of “all-out” sprints does not require previous physical assessment, such as cardiorespiratory exercise testing, which might overcome logistical barriers imposed during social isolation. A common concern raised by exercise specialists is the negative feeling (e.g., reduced enjoyment and displeasure) and feasibility of SIT for sedentary people [61]. However, previous studies showed that reducing the duration of the sprints to ≤10 s might alleviate the negative feelings and increase the exercise enjoyment, as well as expand its applicability [62,63].

The Tabata protocol was originally reported as a type of SIT, involving seven to eight 20-s bouts of high-effort cycling interspersed with 10 s of rest [64]. It is commonly suggested to perform the protocol at a given percentage (110 to 170%) of the intensity associated with maximum oxygen consumption [65,66], which would require specific tests and equipment. However, in the original protocol, exercise was performed at a constant load and was interrupted when the participants were unable to maintain the predetermined intensity [64], which might be more practical. Another practical alternative would be to perform each bout at maximum intensity, as previously suggested [67,68].

Tabata protocol has been used with many different variations that been shown to produce similar physiological adaptations in comparison to traditional aerobic training, but in a time-efficient manner [41]. Among these variations, and noteworthy to the scope of this article, we can highlight the use of body-weight exercises, also called calisthenics (e.g., jumping jacks, mountain climbers, burpees, squats and thrusts) [67,69].

The performance of body-weight IT has been shown to provide similar acute physiological response than those performed in a specialized bicycle, with the advantage to be more enjoyable [70]. Specifically, Tabata protocols using body weight exercises induce similar increases in cardiorespiratory and neuromuscular fitness in comparison to moderate-intensity continuous aerobic training performed on a treadmill [67,69]. The use of body-weight exercises is particularly relevant during social isolation since this allow to perform the protocols anywhere, including at home.

Studies involving body-weight exercises are commonly performed with active healthy people. However, it might be adapted for other populations by increasing work duration and reducing the relative effort (i.e., submaximal intensity) [71,72], changing exercise selection and order based on its complexity, metabolic and neuromuscular demands [73]. Furthermore, practical body-weight exercise with virtual supervision may be an effective alternative during isolation [72], since supervision seems determinant to optimize IT-based programs effects [47]. However, it is important to note that even unsupervised IT programs may induce cardiorespiratory and body composition benefits [74,75].

## 3. Interval Training and Immunity

Although the benefits of regular physical exercise on the immune system are well known [6], the acute effects of aerobic training on immune function depends on how it is done [76,77]. Previous studies suggested that the performance of high-volume or -intensity aerobic training might impair immune response, leading to transitory immunosuppression and increased infection risk [43,44,78,79]. Although, this hypothesis is under debate [80]; it might be advisable to adequately organize IT to avoid these possible negative effects.

The physiological demand of different IT protocols has a different impact on the immune system [81]. Immunosuppression usually occurs after in protocols that result in increased levels of inflammation, metabolic and oxidative stress. A single SIT session involving 30-s bouts of all-out effort have been shown to negatively impact IgA levels [82,83], lymphocytes [84] and neutrophils activity [85] for up to two hours after exercise cessation. Although these effects do not seem to persist for more than a few hours [85,86,87], this might induce an open window for viral infections.

Such negative effects were not found in protocols that have a lower metabolic demand [88,89,90], suggesting that the problem might not be inherent to IT per se, but on how it is performed. Exercise-induced immunosuppression is generally related to high glycolytic activity, high cortisol levels and sympathetic stimulation [76,91]. In fact, IT protocols that induced these metabolic responses have been associated with negative immune outcomes and increased risk of illness [84,85,92,93,94]. Therefore, some strategies might be used to alleviate this responses, like to reduce the duration of the sprints to less than 15 s [95,96,97], reduce the number of sprints [95,98], increase rest duration and perform active rest between sprints [99] or reduce weekly training frequency [100]. Moreover, caution should be taken with training schedule and weekly volume since consecutive days of two daily IT sessions induce acute immunosuppression and increased risk for infection [101,102].

Over medium to long term, regular IT practice can improve immune response regardless of training status [87,103,104,105,106]. Therefore, adapted IT protocols could be a promising strategy to increase immunosurveillance while controlling for acute risks. Additional measures to control the risk of infection, like avoidance the exposure to environments with a high risk of contamination during and for the next two hours after exercise performance should also be considered.

## 4. Practical Recommendations

For SIT, the recommendation is to perform short bouts (≤10 s) of all-out efforts with active rest periods of at least eight times the duration of the bouts. This might help to reduce the negative impact on the immune system and the discomfort associated with SIT. Total volume should involve the accumulation of 60 to 240 s of high-intensity efforts. Tabata protocols can be performed with four to 10 bouts of 20 s interspaced by 10 s of rest. In both cases intensity can be constant aiming to reach exhaustion within the designated number of bouts or with all-out efforts in each bout. It is advisable to avoid exposure to potential contamination during and for the next two hours after exercise, especially in vulnerable populations. Special attention should be given to hygiene and physical distancing (mainly if exercise is performed in outdoor environment and with people that do not live in the same home). If one wants to increase safety, exercise could be performed at home, in stairs, garages or spaces close to home.

The exercise type might involve indoor activities like stair climbing, calisthenics and jumping rope or traditional exercises, like running and cycling. The practitioner might perform the same exercises for all bouts or alternate the exercise performed in each bout. When performing outdoor exercises, it is advisable to choose uncrowded spaces and, when exercising near other people, it is recommended to keep an adequate distance to avoid droplets form air spray. Another important point is to observe hygiene recommendations when sharing equipment or materials. Exercise choice must consider safety and individual limitations to avoid injury risks and the potential hazards of poor exercise performance.

## 5. Final Considerations

Interval training involves many different variables (e.g., effort intensity, effort duration, recovery intensity, recovery duration, number of intervals, number of sets and exercise type) and their combination allows a wide possibility of adaptations [40,42]. The present article discussed specific IT possibilities as potential strategies to overcome the barriers to exercise adoption during social distancing. Cardiorespiratory fitness, metabolic health, mental health and immunological improvements achieved after regular IT performance may contribute to improve general health and reduce the adverse effects and mortality risk due to the COVID-19 outbreak.

Sprint interval training and Tabata protocols performed using minimal equipment in reduced spaces and requiring a minimal time commitment [67,72,107] may be attractive, feasible and safe alternatives to be implemented in face of the social distance measures adopted to control coronavirus dissemination.

However, it is important to understand the characteristics of different IT protocols since different physiological and perceptual responses might impose health risks and detract adherence due to high cardiovascular stress or high neuromuscular demand [108,109]. Additionally, some attention should be given to movement learning, since bad exercise techniques might lead to increased risk for injury, especially hamstrings and knees during running and jumping activities.
